# Comparative effectiveness study of breast-conserving surgery and mastectomy in the general population: A NCDB analysis

**DOI:** 10.18632/oncotarget.5394

**Published:** 2015-10-19

**Authors:** Kai Chen, Jieqiong Liu, Liling Zhu, Fengxi Su, Erwei Song, Lisa K. Jacobs

**Affiliations:** ^1^ Guangdong Provincial Key Laboratory of Malignant Tumor Epigenetics and Gene Regulation, Sun Yat-Sen Memorial Hospital, Sun Yat-Sen University, Guangzhou, China; ^2^ Breast Tumor Center, Sun Yat-Sen Memorial Hospital, Sun Yat-Sen University, Guangzhou, China; ^3^ Departments of Surgery and Oncology, Johns Hopkins Medical Institutions, Baltimore, MD, USA

**Keywords:** breast cancer, breast-conserving surgery, mastectomy

## Abstract

**Purpose:**

Recent studies have revealed that breast-conserving surgery (BCS) with radiotherapy (RT) led to better survival than mastectomy in some populations. We compared the efficacy of BCS+RT and mastectomy using the National Cancer Database (NCDB, USA).

**Methods:**

Non-metastatic breast cancers in the NCDB from 2004–2011 were identified. The Kaplan-Meier method, Coxregression and propensity score analysis were used to compare the overall survival (OS) among patients with BCS+RT, mastectomy alone and mastectomy+RT.

**Results:**

A total of 160,880 patients with a median follow-up of 43.4 months were included. The respective 8-year OS values were 86.5%, 72.3% and 70.4% in the BCS+RT, mastectomy alone and mastectomy+RT group, respectively (*P* < 0.001). After exclusion of patients with comorbidities, mastectomy (alone or with RT) remained associated with a lower OS in N0 and N1 patients. However, the OS of mastectomy+RT was equivalent to BCS+RT in N2–3 patients. Among patients aged 50 or younger, the OS benefit of BCS+RT over mastectomy alone was statistically significant (HR1.42, 95% CI 1.16–1.74), but not clinically significant (<5%) in N0 patients, whereas in N2–3 patients, the OS of BCS+RT was equivalent to mastectomy+RT (85.2% *vs*. 84.8%). The results of the propensity analysis were similar.

**Conclusions:**

BCS+RT resulted in improved OS compared with mastectomy ± RT in N0 and N1 patients. In N2–3 patients, BCS+RT has an OS similar to mastectomy+RT when patients with comorbidities were excluded. Among patients aged 50 or younger, the OS of BCS+RT is equivalent to mastectomy ± RT.

## INTRODUCTION

The long-term survival of early-stage breast cancer patients is equivalent to either breast-conserving surgery (BCS) plus radiation therapy (RT) or modified radical mastectomy, as demonstrated in several prospective randomized controlled trials (RCTs) [1–6]. However, participants in RCT sare highly selected and may not represent the general population. Although RCT scan provide the least biased estimates for treatment comparisons, their results may not correspond to actual clinical situations [7]. In daily routine practice, physicians make decisions based on many uncontrolled factors and apply the results of RCTs to a broader range of patients. Therefore, observational studies are relied on to provide additional information regarding the comparative effectiveness of different treatments in the general population [7]. Abdulkarim, *et al* reported that in T1–2N0M0 triple-negative (TN) patients, modified radical mastectomy without RT significantly increased the risk of local failure compared with BCS+RT [8]. This interesting finding was also observed in Adkins' study [9] but not in that by Zumsteg [10]. In addition, studies using data from national cancer registries [11–13] reported similar findings: that BCS+RT was associated with improved survival compared with mastectomy alone or mastectomy with RT.

In general, the findings from observational studies suffer from selection bias. For example, it is possible that patients who receive BCS+RT are more likely to have fewer comorbidities, which contributes to its superior survival rates. Information about comorbidities was usually unavailable in previous studies [11, 13]. Here, we retrospectively compared the long-term overall survival (OS) between BCS and mastectomy using the National Cancer Database (NCDB). The NCDB is a joint program of the Commission on Cancer of the American College of Surgeons and the American Cancer Society [7]. It includes more than 1,500 commission-accredited cancer programs in the United States and contains detailed tumor pathology information. Additionally, the insurance type, comorbidities (Charlson-Deyo score) and days of inpatient stay were collected, which enable us to identify patients with low/high comorbidities. We hypothesized that by using a large national cohort of breast cancer patients, this analysis would provide additional evidence in the relative effectiveness of the association between local therapy (BCS *vs*. mastectomy) and clinical outcomes. To minimize the influence of “confounding by indications”, we planned to perform subgroup analyses by comorbidities or age. We hypothesized that in patients with no/few comorbidities, or younger age, there would be no differences of OS between BCS and mastectomy.

## RESULTS

We identified 160,880 patients who fit the inclusion and exclusion criteria. The median age was 60 years old, and 59.1%, 34.1% and 6.0% of the patients had AJCC Stage I, II and III disease, respectively. A total of 126,569(78.7%), 26,130(16.2%) and 8,181(5.1%) patients had received BCS+RT, mastectomy alone and mastectomy+RT, respectively. As shown in Table 1, patients in the BCS group were more likely to have private insurance, fewer comorbid diseases (CD scor*e* = 0), lower tumor burden (smaller tumor and/or negative nodes), and fewer hormone receptor-positive diseases. Additionally, BCS patients were wealthier and had a higher education level, and the majority of women (83.9%) received surgery at clinics without inpatient stays after surgery.

**Table 1:** Clinicopathological features of included patients

	Surgery						*P*
	BCS+RT *n* = 126,569		Mastectomy Alone*n* = 26,130		Mastectomy+RT *n* = 8,181		
	*n*	%	*n*	%	*n*	%	
**Facility Type**							
1: Community Cancer Program	13,675	10.8	3,480	13.3	963	11.8	<0.001
2: Comprehensive Community Cancer Program	76,163	60.2	16,039	61.4	4,850	59.3	
3: Academic/Research Program	36,500	28.8	6,582	25.2	2,358	28.8	
9: Other Specified Types Of Cancer Programs	231	0.2	29	0.1	10	0.1	
**Primary Payor**							
0: Not Insured	2,048	1.6	632	2.4	307	3.8	<0.001
1: Private Insurance	78,123	61.7	11,151	42.7	4,651	56.9	
2: Medicaid	6,573	5.2	1,963	7.5	898	11.0	
3: Medicare	38,745	30.6	12,173	46.6	2,253	27.5	
4: Other Government	1,080	0.9	211	0.8	72	0.9	
**City Type**							
Metropolitan	109,539	86.5	21,269	81.4	6,807	83.2	<0.001
Urban	15,167	12.0	4,190	16.0	1,223	14.9	
Rural	1,863	1.5	671	2.6	151	1.8	
**Distance**							
<10_Miles	72,677	57.4	14,326	54.8	4,447	54.4	<0.001
>10_Miles	53,892	42.6	11,804	45.2	3,734	45.6	
**Median Income Quartiles 2008–2012**							
<$47999	43,364	34.3	11,675	44.7	3,520	43.0	<0.001
$48000+	83,205	65.7	14,455	55.3	4,661	57.0	
**Percent No High School Degree 2008–2012**							
>=13%	44,585	35.2	11,813	45.2	3,635	44.4	<0.001
<13%	81,984	64.8	14,317	54.8	4,546	55.6	
**Age Group**							
<=60	68,492	54.1	10,463	40.0	5,008	61.2	<0.001
>60	58,077	45.9	15,667	60.0	3,173	38.8	
**Race**							
White	109,050	86.2	21,731	83.2	6,525	79.8	<0.001
African American	12,782	10.1	2,946	11.3	1,173	14.3	
Others	4,737	3.7	1,453	5.6	483	5.9	
**Charlson-Deyo Score**							
0	109,623	86.6	20,216	77.4	6,785	82.9	<0.001
1	14,559	11.5	4,662	17.8	1,129	13.8	
2	2,387	1.9	1,252	4.8	267	3.3	
**T-Stage**							
T1	98,660	77.9	15,001	57.4	2,841	34.7	<0.001
T2	27,909	22.1	11,129	42.6	5,340	65.3	
**N-Stage**							
N0	98,236	77.6	17,756	68.0	1,236	15.1	<0.001
N1	23,480	18.6	7,114	27.2	3,276	40.0	
N2	3,776	3.0	938	3.6	2,622	32.0	
N3	1,077	0.9	322	1.2	1,047	12.8	
**Stage**							
I	82,728	65.4	11,725	44.9	689	8.4	<0.001
II	39,022	30.8	13,148	50.3	3,845	47.0	
III	4,819	3.8	1,257	4.8	3,647	44.6	
**Grade**							
I	30,644	24.2	4,213	16.1	744	9.1	<0.001
II	54,309	42.9	11,114	42.5	3,180	38.9	
III	41,616	32.9	10,803	41.3	4,257	52.0	
**Estrogen Receptor**							
Negative	24,873	19.7	7,218	27.6	2,205	27.0	<0.001
Positive	101,696	80.3	18,912	72.4	5,976	73.0	
**Progesterone Receptor**							
Negative	36,308	28.7	9,812	37.6	3,047	37.2	<0.001
Positive	90,261	71.3	16,318	62.4	5,134	62.8	
**Laterality**							
Right	62,677	49.5	12,643	48.4	4,018	49.1	<0.001
Left	63,892	50.5	13,487	51.6	4,163	50.9	
**Primary Site**							
Central	4,554	3.6	1,907	7.3	550	6.7	<0.001
LIQ	8,133	6.4	1,669	6.4	415	5.1	
LOQ	9,714	7.7	2,063	7.9	694	8.5	
UIQ	308	0.2	162	0.6	38	0.5	
UOQ	35,575	28.1	8,962	34.3	2,977	36.4	
Nipple	17,925	14.2	3,014	11.5	650	7.9	
Others	50,360	39.8	8,353	32.0	2,857	34.9	
**Lymphovascular Invasion**							
Negative	37,013	29.2	6,293	24.1	1,286	15.7	<0.001
Positive	7,562	6.0	1,790	6.9	1,394	17.0	
Unknown	81,994	64.8	18,047	69.1	5,501	67.2	
**Chemotherapy**							
No	68,989	54.5	14,128	54.1	1,156	14.1	<0.001
Yes	57,580	45.5	12,002	45.9	7,025	85.9	
**Days Of Inpatient Stay**							
0	106,199	83.9	5,537	21.2	2,126	26.0	<0.001
1	14,138	11.2	13,287	50.8	3,965	48.5	
>1	6,232	4.9	7,306	28.0	2,090	25.5	

BCS, breast-conserving surgery; RT, Radiation therapy; LIQ, lower-inner quadrant; LOQ, lower-outer quadrant; UIQ, Upper-inner quadrant; UOQ, Upper-outer quadrant; NS, non-significant;

### Survival benefit of BCS over mastectomy in the entire study cohort

**Figure 1:**
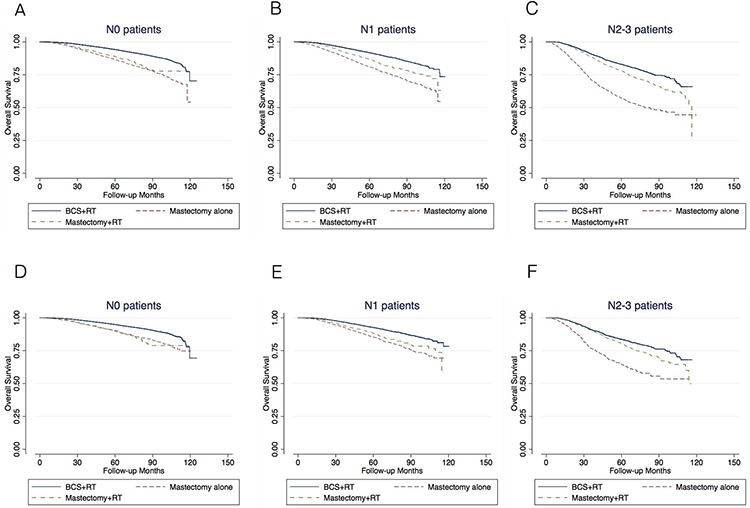
Kaplan-Meier survival analysis of the entire population A–C. and in patients with “Less/No comorbid conditions” D–F. Analysis were performed separately in N0 (A, D), N1 (B, E) and N2–3 (C, F) patients

With a median follow-up of 43.4 months, the respective 5-year and 8-year OS values were 93.2% and 86.5% in the BCS+RT group, 83.5% and 72.3% in the mastectomy-alone group, and 83.0% and 70.4% in the mastectomy+RT group, respectively (log-rank test; *P* < 0.001). Univariate (Supplementary Table 1) and multivariate analyses (Supplementary Table 2A) revealed that mastectomy (alone or with RT) was significantly associated with a lower 5-year and 8-year overall survival in patients with N0, N1 and N2–3 disease compared with BCS+RT (Figure 1A–1C, Table 2).

**Table 2:** Survival benefit of BCS+RT over mastectomy+/−RT varied across patients with different comorbid diseases or age

Features		*N*	Overall survival %		Cox-regerssion**				Post-mastectomy OS benefit*	
					Unadjusted		Adjusted			
			5-year	8-year	HR (95%CI)	*P*	HR (95%CI)	*P*	5-year	8-year
**All patients#**										
N0	BCS+RT	98,236	94.2%	88.0%	1		1			
	Mastectomy_alone	17,756	86.5%	75.8%	2.39(2.27–2.52)	<0.001	1.40(1.31–1.50)	<0.001	2.34%	1.94%
	Mastectomy+RT	1,236	88.9%	77.8%	1.96(1.64–2.35)	<0.001	1.52(1.26–1.83)	0.001		
N1	BCS+RT	23,480	91.8%	83.8%	1		1			
	Mastectomy_alone	7,114	81.0%	68.8%	2.44(2.26–2.63)	<0.001	1.44(1.31–1.58)	<0.001	5.71%	7.25%
	Mastectomy+RT	3,276	86.7%	76.1%	1.64(1.46–1.85)	<0.001	1.33(1.17–1.51)	<0.001		
N2-3	BCS+RT	4,853	82.9%	73.3%	1		1			
	Mastectomy_alone	1,260	57.1%	46.6%	3.01(2.67–3.39)	<0.001	1.64(1.42–1.88)	<0.001	21.00%	16.87%
	Mastectomy+RT	3,669	78.1%	63.4%	1.34(1.20–1.49)	<0.001	1.12(1.00–1.26)	0.052		
**Patiens with less/no comorbid diseases ##**										
N0	BCS+RT	81,893	94.9%	89.3%	1		1			
	Mastectomy_alone	10,550	89.9%	81.5%	2.01(1.86–2.17)	<0.001	1.38(1.28–1.49)	<0.001	0.39%	−2.62%
	Mastectomy+RT	900	90.3%	78.9%	2.00(1.60–2.52)	<0.001	1.67(1.33–2.10)	<0.001		
N1	BCS+RT	18,742	92.6%	85.5%	1		1			
	Mastectomy_alone	3,844	85.3%	73.6%	2.07(1.86–2.31)	<0.001	1.41(1.26–1.58)	<0.001	2.95%	4.80%
	Mastectomy+RT	2,079	88.2%	78.4%	1.66(1.42–1.93)	<0.001	1.41(1.21–1.65)	<0.001		
N2-3	BCS+RT	3,775	83.6%	74.9%	1		1			
	Mastectomy_alone	653	64.8%	53.5%	2.49(2.11–2.94)	<0.001	1.52(1.27–1.82)	<0.001	15.92%	13.03%
	Mastectomy+RT	2,206	80.7%	66.6%	1.22(1.07–1.40)	0.003	1.12(0.97–1.28)	0.126		
**Patiens with age<=50 ###**										
N0	BCS+RT	22,638	96.7%	94.0%	1		1			
	Mastectomy_alone	3,106	93.7%	89.3%	2.01(1.70–2.38)	<0.001	1.42(1.16–1.74)	0.001	−0.76%	−4.55%
	Mastectomy+RT	449	92.9%	84.7%	2.31(1.59–3.36)	<0.001	1.70(1.15–2.50)	0.007		
N1	BCS+RT	6,857	93.6%	88.9%	1		1			
	Mastectomy_alone	1,450	90.2%	82.8%	1.49(1.21–1.83)	<0.001	1.13(0.90–1.43)	0.292	1.38%	−1.50%
	Mastectomy+RT	1,242	91.5%	81.3%	1.50(1.20–1.88)	0.001	1.23(0.96–1.57)	0.101		
N2-3	BCS+RT	1,615	85.2%	77.7%	1		1			
	Mastectomy_alone	247	73.5%	68.1%	1.81(1.34–2.43)	<0.001	1.20(0.87–1.67)	0.27	11.28%	7.42%
	Mastectomy+RT	1,097	84.8%	75.5%	1.06(0.86–1.31)	0.587	0.92(0.72–1.17)	0.514		
**Patiens with age>50 ###**										
N0	BCS+RT	75,598	93.4%	85.8%	1		1			
	Mastectomy_alone	14,650	84.9%	72.5%	2.34(2.21–2.47)	<0.001	1.40(1.31–1.51)	<0.001	1.60%	0.34%
	Mastectomy+RT	787	86.5%	72.9%	2.11(1.71–2.60)	<0.001	1.47(1.19–1.82)	0.004		
N1	BCS+RT	16,623	90.9%	81.4%	1		1			
	Mastectomy_alone	5,664	78.4%	64.8%	2.54(2.33–2.75)	<0.001	1.51(1.37–1.67)	<0.001	5.05%	7.92%
	Mastectomy+RT	2,034	83.5%	72.8%	1.79(1.56–2.05)	<0.001	1.38(1.19–1.60)	<0.001		
N2-3	BCS+RT	3,238	81.7%	70.7%	1		1			
	Mastectomy_alone	1,013	52.8%	40.1%	3.23(2.83–3.68)	<0.001	1.86(1.60–2.18)	<0.001	22.29%	17.93%
	Mastectomy+RT	2,572	75.1%	58.0%	1.43(1.26–1.61)	<0.001	1.22(1.06–1.40)	0.005		

* Post-mastectomy RT benefit=cumulative survival rate of mastectomy+RT - cumulative survival rate of mastectomy alone

** For multivariable anslysis, facility type, primary payor, city type, distance to hospital, median income, percentage of no high school degree, age, race, Charlson-Deyo score, T-stage, N-stage, Grade, estrogen receptor, progesterone receptor, primary site, lymphovascular invasion, radiation therapy, chemotherapy, days of inpatient stay and surgery were incorported into the full model. In subgroup analysis, the respective variables that had been used for stratification were excluded as indicated.

# N-stage were excluded from the full model of multivariate analysis.

## N-stage, days of inpatient stay and Charlson-Deyo score were excluded from the full model of multivariate analysis

### N-stage and age were excluded from the full model of multivariate analysis

BCS, breast-conserving surgery; RT, radiation therapy; HR, hazard ratio; CI, confidence interval;

### Survival benefit of BCS over mastectomy in patients with fewer/no comorbidities

**Figure 2:**
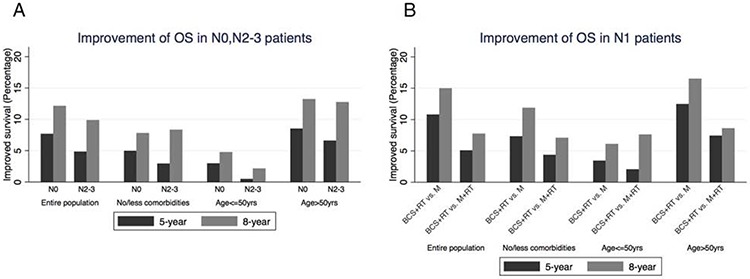
A. Improvement of OS in N0, N2–3 patients. The benefit of 5-year and 8-year OS was calculated by comparing BCS+RT with mastectomy alone in N0 patients, and with mastectomy+RT in N2–3 patients **B.** Improvement of OS in N1 patients. The survival benefit of BCS+RT over mastectomy alone or with RT was shown as indicated. BCS, breast-conserving surgery; M, Mastectomy; RT, radiation therapy.

A total of 124,642 patients with a CD score of 0 and days of inpatient stay ≤1 were considered to have fewer/no comorbid diseases and were included in this analysis (Table 2, Figure 2). In N0 patients, BCS+RT (vs. mastectomy alone) increased the 5-year and 8-year OS by 5.0% and 7.8%, respectively. After adjustment, mastectomy alone (*vs*. BCS+RT) was significantly associated with a lower OS (HR 1.38, 95% CI 1.28–1.49) (Supplementary Table 2B). In N1 patients, mastectomy (alone or with RT) was significantly correlated with a lower OS (Table 2, Figure 2B). However, among N2–3 patients, mastectomy+RT (*vs*. BCS+RT) was no longer associated with a lower OS after adjustment (HR 1.12, 95% CI 0.97–1.28). The 5-year OS benefit of BCS+RT over mastectomy+RT was less than 5% (Figure 2A).

### Survival benefit of BCS over mastectomy varied by age

Among patients aged 50 or younger, the 5-year and 8-year survival benefit of BCS+RT (*vs*. mastectomy alone or with RT) was significantly lower than that of patients with age ≥50yrs (Figure 2, 3 Supplementary Table 2C). BCS+RT over mastectomy alone was statistically significant (HR 1.42, 95% CI 1.16–1.74) but not clinically significant (5-year: 2.9%; 8-year: 4.8%) in N0 patients. In N1 and N2–3 patients, BCS+RT over mastectomy+RT was not significantly correlated with an improved OS (Table 2 Supplementary Table 2C). The survival benefit of BCS+RT *vs*. mastectomy (alone or with RT) in patients older than 50 were similar to the entire population (Table 2, Figure 2, 3 Supplementary table 2d).

**Figure 3:**
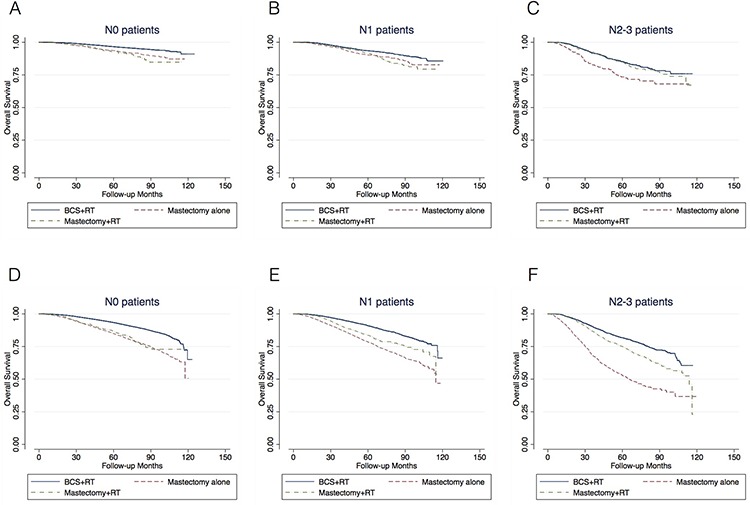
Kaplan-Meier survival analysis of the patients with age ≤50 A–C. and age > 50 D–F. respectively Analysis was performed separately in N0 (A, D), N1 (B, E) and N2–3 (C, F) patients

### Propensity score analysis of OS

We used propensity scores to create strata in which the possibility of having a specific treatment (BCS+RT *vs*. mastectomy ± RT) was similar for all patients in the same strata, regardless of their actual received treatment. Using a Cox-regression model stratified by propensity score quintile, we found that mastectomy alone (*vs*. BCS+RT) was associated with a worse OS in N0 (HR 1.75, 95% CI 1.66–1.85) and N1 patients(HR 1.73, 95% CI 1.60–1.88). Mastectomy+RT *vs*. BCS+RT was correlated with a lower OS in N1 patients (HR 1.24, 95% CI 1.10–1.39), but not in N2–3 patients (HR 1.09, 95% CI 0.98–1.21).

### Survival benefit of post-mastectomy radiotherapy (PMRT)

In the entire cohort, the 5-year OS benefit of PMRT (mastectomy+RT *vs.* mastectomy alone) was 2.3%, 5.7% and 21.0% in N0, N1 and N2–3 patients, respectively (Table 2, Figure 1A-1C). After the exclusion of patients with comorbid conditions, the respective OS benefit of PMRT was less than 5% in N0 and N1 patients, and 15.9% in N2–3 patients (Table 2, Figure 1D-1F). Among patients aged 50 or younger, there was no benefit of PMRT in N0 and N1 patients, while in N2–3 patients, the OS benefit of PMRT was 11.3% (Table 2, Figure 3).

## DISCUSSION

### Recent studies of the comparative effectiveness of BCS+RT *vs*. mastectomy

Randomized controlled trials had demonstrated that BCS has an equivalent long-term survival to mastectomy [3, 4, 6, 14–18] in early-stage breast cancer patients. The comparative effectiveness of BCS+RT *vs*. mastectomy in non-clinical-trial population has been investigated in administrative, observational database. Hwang, *et al* [13] identified 112,154 stage I and II breast cancer patients from the large population-based prospective California Cancer Registry. The authors observed that women who underwent BCS+RT had improved breast cancer specific survival(BCSS) and OS compared with those treated by mastectomy, and the survival benefit of BCS+RT was greater among “≥50yrs & HR+” women. Brooks, *et al* [19] used the instrumental variable method to study the SEER-Medicare database and reported that higher mastectomy rates were associated with reduced survival. Other studies using data from national cancer registries in Norway [12], Canada [20] and the US [11, 21] reported similar findings (Table 3).

**Table 3:** Comparative effectiveness studies comparing clinical outcomes of patients receiving BCS and mastectomy in non-clinical trial population

Article	Data source	Years of diagnosis	Inclusion criteria	Median follow-up (months)	Surgery group	Sample size	Outcomes			
							5-year %	10-year %	5-Year difference* %	10-year difference* %
Hwang et al.	California Cancer Registry, US	1990–2004	T1/T2, Stage I-II,	110.6	BCS+RT	61,777	CSS:89.3	N/A	N/A	N/A
					Mastectomy alone	50,383	OS:94.4			
Agarwal et al	SEER, US	1998–2008	Tumor size<4cm or positive lymph nodes<3	N/A	BCS+RT	92,671	CSS:97	CSS:94	CSS: 3–7	CSS: 4–11
					Mastectomy alone	34,999	CSS:94	CSS:90		
					Mastectomy+RT	4,479	CSS:90	CSS:83		
Zumsteg et al.	Memorial Sloan-Kettering	1999–2008	T1-T2N0, TNBC	76.4	BCS+RT	448	LRFS:95	N/A	LRFS: 1.2	N/A
					Mastectomy alone	198	LRFS:94		DMFS:	N/A
Fisher et al.	Alberta Cancer Registry, Canada	2002–2010	Stage I-III	50.4	BCS alone	805	OS:74	N/A	OS:11CSS (Stage I):98.2	N/A
					BCS+RT	5,722		OS:94		
					Mastectomy+/−RT	8,412		OS:83		
Hartmann-Johnsen et al.	Cancer Registry of Norway	1998–2008	T1–2N0–1M0	N/A	BCS+RT	8,065	CSS:97	N/A	CSS: 9 OS: 15	N/A
					Mastectomy+/−RT	4,950	CSS:88			
Abdulkarim et al.	Alberta Cancer Registry, Canada	1998–2008	T1-T2N0, TNBC	86.4	BCS+RT	319	LRFS:94	N/A	LRFS:7–9 OS:5–19	N/A
					Mastectomy alone	287	LRFS:85			
					Mastectomy+RT	162	LRFS:87			
Adkins et al.	M. D. Anderson Cancer Center, US	1980–2007	Stage I-III, TNBC	62	BCS+/−RT	651	LRFS:76	N/A	LRFS: 5 DMFS:14	N/A
					Mastectomy+/−RT	674	LRFS:71			
Onitilo et al.	Marshfield Clinic, US	1994–2012	Stage 0-IV	67	BCS+/−RT	3,340	OS:90.5	OS:78.4	OS: 6.3	N/A
					Mastectomy+/−RT	1,995	OS:84.2	OS:62.8		

* Five-year or ten-year differences were calculated as the survival rate of BCS - survival rate of mastectomy.

BCS, breast-conserving surgery; RT, radiotherapy, LRR, local-recurrence-free survival; DMFS, distant-metastasis-free survival; OS, overall survival; CSS, cancer-specific survival; N/A, not available; TNBC, triple negative breast cancer; SEER, Surveillance, epidemiology and end results.

Fisher *et al* [20]. used data from Alberta Cancer Registry (Canada) and showed that the survival benefit of BCS+RT *vs*. mastectomy was less significant in stage I patients, than in stage II or III patients. The underlying reason may due to the low risk of relapse events in stage I patients, rendering the survival advantages of BCS+RT less likely to be noted. However, there were two limitations of their study: 1) They did not distinguish patients who received PMRT from those who did not. Additionally, they stratified the analysis by cancer stage, rather than N-stage. Thus, in the stage II strata that contained both node-negative and node-positive patients, the comparison of BCS *vs*. mastectomy was significantly confounded by the unknown PMRT status. 2) The proportion of patients with non-standard treatment (e.g. PMRT in N0 patients after mastectomy, or no PMRT in N2–3 patients after mastectomy) in their mastectomy group was unknown. Therefore, the accuracy and generalizability of their results were influenced.

In our study, we stratified the survival analysis by N-stage. We compared BCS+RT *vs*. mastectomy alone, BCS+RT *vs*. mastectomy alone *vs*. mastectomy+RT, BCS+RT *vs*. mastectomy+RT in N0, N1 and N2 patients, respectively. We believe that our design is clearer in purpose and more informative for clinical practices. Our study showed that in N0 patients that PMRT is not recommended, BCS+RT had better OS than mastectomy alone; whereas in N2–3 patients that PMRT is routinely performed, BCS+RT has equivalent OS to mastectomy+RT after adjustment (Table 2, Figure 1). These results suggested the importance of RT in the comparison of BCS and mastectomy using administrative database.

### The impact of comorbid conditions and age

Confounding by indication is the major limitation for most retrospective studies. Specifically, patients with fewer comorbid conditions are more likely to receive BCS+RT and these patients are more likely to have better OS than those with many comorbidites. Land. *et al* [22] determined that patients with more comorbid conditions were more likely to die from breast cancer as well as other causes, using data from the Danish Breast Cancer Cooperative Group Registry. A study by Hwang et al [13] using the California Cancer Registry database showed that patients who received BCS+RT were less likely to die from cardiovascular disease, or chronic lower respiratory diseases. They inferred that mastectomy patients were more likely to have comorbid diseases, which may have influenced the surgical decision. Hence, we performed subgroup analysis by excluding patients with severe comorbidities. In the NCDB, the Charlson-Deyo (CD) score has been used to describe comorbid conditions. We defined patients with CD score = 0 (no comorbid conditions) and days of inpatient stay ≤1 as “low/no comorbidity” patients. In this subgroup of women, the 5-year OS benefit of BCS+RT was 5.0% in N0 patients (*vs.* mastectomy alone), and 2.9% in N2–3 patients (*vs*. mastectomy+RT). Compared with the results derived from our entire study population, the survival benefit of BCS+RT over mastectomy (alone or with RT) was decreased, suggesting that the significant survival benefit of BCS+RT in previous studies might be partially due to comorbid conditions. Subgroup analysis in patients with comorbidities was not performed, because the effect of “confounding by indication” may be more significant in these patients.

Survival analysis was also stratified by age. As shown in Table 2 and Figure 3, the 5-year and 8-year OS benefit of BCS+RT was less than 5% in patients aged 50 or younger, regardless of nodal status. The survival advantages of BCS+RT in patients older than 50 were similar to the entire population. Our result is consistent with previous studies. A population-based study by Cao *et al* [23] found that 965 patients aged 20 to 39 years with breast cancer treated from 1989 to 2003 showed no difference in the 15-year rates of BCSS. Similar results were observed in other studies [24, 25]. The findings from these studies, together with ours, reveal the oncological safety of BCS+RT, particularly in young patients.

### Benefit of less extensive surgery: is it possible?

More extensive surgery may theoretically lead to better or at least equivalent outcomes as less extensive surgery in cancer treatment. Is it possible that the opposite may be true? Studies in animal models have suggested that the surgical trauma of normal tissue promotes the implantation or growth of circulating tumor cells [26–29]. Thus, with mastectomy, would a larger wound produce more cytokines to activate the distant dormant tumor cells? There is evidence from clinical studies indicating such a possibility. A randomized controlled trial published in *Lancet* [30] compared the efficacy of laparoscopy-assisted colectomy and open colectomy for the treatment of non-metastatic colon cancer in terms of tumor relapse and CSS. With lesser surgical treatment, laparoscopy-assisted colectomy had a significantly higher CSS. Further studies of these hypothesis and exploration of the underlying mechanisms are needed.

### Benefit of PMRT

The benefit of PMRT in patients with N1 disease is controversial. A subgroup analysis of DBCG 82 randomized trials [31] suggested a similar OS benefit of RT in patients with N1 and N2–3 disease. Likewise, an EBCTCG meta-analysis using individual data from 22 trials [32, 33] revealed that PMRT reduced the rate of mortality in breast cancer patients, which was independent of the number of lymph nodes involved. The National Comprehensive Cancer Network (NCCN) guidelines [34] also recommend strong consideration of PMRT in N1 patients. However, our study demonstrated that PMRT was associated with an improved 5-year OS in N2–3 patients rather than N0 or N1 patients (Table 2). Our study was not the first to question the need for PMRT in N1 patients. He, *et al* [35] reported that, in patients with T1–2 and N1 diseases, the survival benefit of PMRT was present only in high-risk patients. Huo, *et al* [36]analyzed 93,793 and 36,299 T1–2N1 breast cancer patients in the NCDB and SEER database and showed that PMRT improved OS among patients with 3 positive nodes or 2 positive nodes with tumors 2–5 cm in size, but had no benefit in patients with 1 positive node or 2 positive nodes with tumor ≤2 cm. McBride, *et al* [37] also observed that the benefit of PMRT on local control was highly dependent on the era of treatment: PMRT reduced the risk of 5-year LRR in patients treated in an early era (1978–1998), but not in those treated in a later era (2000–2007), when the use of sentinel lymph node surgery, taxane chemotherapy, and aromatase inhibitors was routine. Taken together, we may reconsider the benefit of PMRT on OS among N1 breast cancer patients. Further randomized controlled studies addressing this issue are warranted.

### Limitations

In this observational study, we were not able to retrieve information such as multifocality, EIC, HER2 status, BRCA status, body-mass index, detailed chemotherapy, endocrine therapy, targeted therapy and cancer-specific survival (CSS) from the NCDB. Thus, the selection biases cannot be completely diminished, although we used propensity score analysis. 1) BRCA mutation-positive patients would tend to receive mastectomy. However, a recent study showed that the 10-year OS is similar between patients with or without the BRCA mutation [38]. In addition, HER2 status is not expected to be associated with the choice of surgery (mastectomy *vs*. BCS). Therefore, the failure to match for BRCA status or HER2 status would not have a significant impact on our analysis. 2) Patients with positive margins, who were converted to mastectomy from BCS, were likely associated with multifocal DCIS or EIC. Hence, the inability to match for multifocality is a major limitation of this study. 3). Lack of information of BCSS in the NCDB is another limitation of our study. However, a summary of previous studies [8, 9, 12, 20] showed that the survival benefit of BCS+RT (over mastectomy) on BCSS was similar to that of OS (Table 3). Thus, we think that OS is also a valid endpoint for our study. 4) We are not able to ensure that the chemotherapy treatments were completely comparable between groups.

## MATERIALS AND METHODS

### Data collection

We searched the NCDB registry data between 2004 and 2011 and identified female patients with a T1–2N0–3M0 infiltrating duct breast carcinoma diagnosis. The following information was collected: facility type (community cancer program, comprehensive community cancer program, academic/research program, other program); primary payer (not insured, private insurance, Medicaid, Medicare, other government); city type (metropolitan, urban, rural); distance to hospital; median income of the state/area (2008–2012) where the patient lived; the percentage of population without a high school degree of the state/area where the patient lived; age; race; T stage; N stage; AJCC stage; laterality (left or right breast); estrogen receptor (ER) status; progesterone receptor (PR) status; grade; surgery of primary site; radiation; survival (months); vital status; primary site (LIQ, lower-inner quadrant; LOQ, lower-outer quadrant; UIQ, Upper-inner quadrant; UOQ, Upper-outer quadrant; nipple; others); days of inpatient stay; Charlson-Deyo (CD) score; lymphovascular invasion status; chemotherapy(administered as first course treatment); and hormonal therapy (administered as first course treatment). For the breast surgery code, we used the NAACCR item #1290 coding rules (breast, C50.0–C50.9,http://ncdbpuf.facs.org/?q=content/breast). We defined codes 20–24 as BCS and codes 41 and 51 as mastectomy.

The data within the NCDB are rendered anonymous; therefore, the study was exempt from the Johns Hopkins Medicine Institutional Review Board review, and no consent was required.

### Inclusion and exclusion criteria

We had rigorous inclusion and exclusion criteria for patient selection. All of the included patients met the following inclusion and exclusion criteria.

### Inclusion criteria

1. Female, T1–2N0–3M0 breast cancer patients, diagnosis after 2004.
2. Infiltrating ductal breast carcinoma (histology coding 8500) with confirmed pathology diagnosis.
3. Patients with grossly and microscopically negative margins.

### Exclusion criteria

1. Patients with previous diagnosis of breast cancer or any malignant tumors (In this study, patients with the sequence number code of 00 or 01 were included)
2. Patients with pure DCIS or stage 0 disease.
3. Patients with unknown information for any of the included variables, except for lymphovascular invasion.
4. Patients with bilateral breast cancers.
5. Patients with a pathological tumor size larger than 5 cm.
6. Patients with a history of RT.
7. Patients who did not receive RT after BCS.
8. Patients who received hormone therapy for ER- and PR- disease.
9. Patients who did not receive hormone therapy for ER+ or PR+ disease.

### Statistical analyses

The chi-square test was used to compare the demographic and clinicopathological features of patients among three groups: the BCS + RT group; the mastectomy-alone group; and the mastectomy + RT group. Kaplan-Meier survival analysis and an unadjusted Cox proportional hazards model were used to compare the overall survival (OS) of patients who received the different treatments, as a univariate approach. Significant factors revealed by univariate analysis were incorporated into multivariate analysis (adjusted Cox proportional hazards model) and the model was used through out the entire study. In subgroup analysis, we planned to assess the effect of surgery type on OS in patients stratified by N-stages, comorbidities and ages. The variables that were used for stratification were not included in the multivariate model during subgroup analysis.

Propensity analysis was used to compare treatment groups within strata to minimize selection bias or a lack of covariate balance. We considered that age, facility type, primary payer, primary sites, income, urban type, education, distance to hospital, CD score, race, tumor grade, ER, PR, T stage and N stage were all potential determinants for the choice of surgery. Thus, all of these variables were included as conditioning variables in a logistic model, with surgery type (BCS+RT *vs*. mastectomy alone or RT) as the outcome variable. The propensity score was then calculated as the probability of receiving a mastectomy (alone or with RT) for each individual. We stratified the patients into quintiles, in which patients had a similar likelihood of having received a given treatment. Using the Cox model, we estimated the effect of different treatments (BCS+RT, mastectomy alone or mastectomy+RT) on OS, with the baseline survival function varied across strata by including quintiles of the propensity scores as stratification variables.

All *P* values are two sided. *P* values <0.05 were considered statistically significant. A survival difference larger than 5% was considered clinically significant. Statistical analyses were performed using Stata/MP, version 13.0 (StataCorp LP, College Station, TX, USA).

## CONCLUSIONS

This analysis of a large national cohort of patients demonstrates that BCS+RT provides a superior OS to mastectomy (alone or with RT) in N0 and N1 patients, regardless of comorbid conditions. In N2–3 patients, the survival benefit of BCS+RT (*vs*. mastectomy+RT) was eliminated when patients with comorbid conditions were excluded. Among patients aged 50 or younger, the BCS+RT OS is equivalent to mastectomy (alone or with RT). Because mastectomy is significantly more invasive than BCS+RT, we recommend greater efforts at educating patients to undergo BCS+RT rather than mastectomy in our routine practice, particularly for low-risk N0/N1 women.

## SUPPLEMENTARY TABLES













## References

[1] Blichert-Toft M, Nielsen M, During M, Moller S, Rank F, Overgaard M, Mouridsen HT (2008). Long-term results of breast conserving surgery vs. mastectomy for early stage invasive breast cancer: 20-year follow-up of the Danish randomized DBCG-82TM protocol. Acta Oncol.

[2] Dewar JA, Arriagada R, Benhamou S, Benhamou E, Bretel JJ, Pellae-Cosset B, Marin JL, Petit JY, Contesso G, Sarrazin D (1995). Local relapse and contralateral tumor rates in patients with breast cancer treated with conservative surgery and radiotherapy (Institut Gustave Roussy 1970-1982). IGR Breast Cancer Group. Cancer.

[3] Fisher B, Anderson S, Bryant J, Margolese RG, Deutsch M, Fisher ER, Jeong JH, Wolmark N (2002). Twenty-year follow-up of a randomized trial comparing total mastectomy, lumpectomy, and lumpectomy plus irradiation for the treatment of invasive breast cancer. N Engl J Med.

[4] Litiere S, Werutsky G, Fentiman IS, Rutgers E, Christiaens MR, Van Limbergen E, Baaijens MH, Bogaerts J, Bartelink H (2012). Breast conserving therapy versus mastectomy for stage I-II breast cancer: 20 year follow-up of the EORTC 10801 phase 3 randomised trial. Lancet Oncol.

[5] Simone NL, Dan T, Shih J, Smith SL, Sciuto L, Lita E, Lippman ME, Glatstein E, Swain SM, Danforth DN, Camphausen K (2012). Twenty-five year results of the national cancer institute randomized breast conservation trial. Breast Cancer Res Treat.

[6] Veronesi U, Cascinelli N, Mariani L, Greco M, Saccozzi R, Luini A, Aguilar M, Marubini E (2002). Twenty-year follow-up of a randomized study comparing breast-conserving surgery with radical mastectomy for early breast cancer. N Engl J Med.

[7] Hershman DL, Wright JD (2012). Comparative effectiveness research in oncology methodology: observational data. J Clin Oncol.

[8] Abdulkarim BS, Cuartero J, Hanson J, Deschenes J, Lesniak D, Sabri S (2011). Increased risk of locoregional recurrence for women with T1-2N0 triple-negative breast cancer treated with modified radical mastectomy without adjuvant radiation therapy compared with breast-conserving therapy. J Clin Oncol.

[9] Adkins FC, Gonzalez-Angulo AM, Lei X, Hernandez-Aya LF, Mittendorf EA, Litton JK, Wagner J, Hunt KK, Woodward WA, Meric-Bernstam F (2011). Triple-negative breast cancer is not a contraindication for breast conservation. Ann Surg Oncol.

[10] Zumsteg ZS, Morrow M, Arnold B, Zheng J, Zhang Z, Robson M, Traina T, McCormick B, Powell S, Ho AY (2013). Breast-conserving therapy achieves locoregional outcomes comparable to mastectomy in women with T1-2N0 triple-negative breast cancer. Ann Surg Oncol.

[11] Agarwal S, Pappas L, Neumayer L, Kokeny K, Agarwal J (2014). Effect of breast conservation therapy vs mastectomy on disease-specific survival for early-stage breast cancer. JAMA surgery.

[12] Hartmann-Johnsen OJ, Karesen R, Schlichting E, Nygard JF (2015). Survival is Better After Breast Conserving Therapy than Mastectomy for Early Stage Breast Cancer: A Registry-Based Follow-up Study of Norwegian Women Primary Operated Between 1998 and 2008. Ann Surg Oncol.

[13] Hwang ES, Lichtensztajn DY, Gomez SL, Fowble B, Clarke CA (2013). Survival after lumpectomy and mastectomy for early stage invasive breast cancer: the effect of age and hormone receptor status. Cancer.

[14] Poggi MM, Danforth DN, Sciuto LC, Smith SL, Steinberg SM, Liewehr DJ, Menard C, Lippman ME, Lichter AS, Altemus RM (2003). Eighteen-year results in the treatment of early breast carcinoma with mastectomy versus breast conservation therapy: the National Cancer Institute Randomized Trial. Cancer.

[15] van Dongen JA, Bartelink H, Fentiman IS, Lerut T, Mignolet F, Olthuis G, van der Schueren E, Sylvester R, Tong D, Winter J (1992). Factors influencing local relapse and survival and results of salvage treatment after breast-conserving therapy in operable breast cancer: EORTC trial 10801, breast conservation compared with mastectomy in TNM stage I and II breast cancer. Eur J Cancer.

[16] Sarrazin D, Le MG, Arriagada R, Contesso G, Fontaine F, Spielmann M, Rochard F, Le Chevalier T, Lacour J (1989). Ten-year results of a randomized trial comparing a conservative treatment to mastectomy in early breast cancer. Radiother Oncol.

[17] Blichert-Toft M, Rose C, Andersen JA, Overgaard M, Axelsson CK, Andersen KW, Mouridsen HT (1992). Danish randomized trial comparing breast conservation therapy with mastectomy: six years of life-table analysis. Danish Breast Cancer Cooperative Group. J Natl Cancer Inst Monogr.

[18] Voogd AC, Nielsen M, Peterse JL, Blichert-Toft M, Bartelink H, Overgaard M, van Tienhoven G, Andersen KW, Sylvester RJ, van Dongen JA (2001). Differences in risk factors for local and distant recurrence after breast-conserving therapy or mastectomy for stage I and II breast cancer: pooled results of two large European randomized trials. J Clin Oncol.

[19] Brooks JM, Chrischilles EA, Landrum MB, Wright KB, Fang G, Winer EP, Keating NL (2012). Survival implications associated with variation in mastectomy rates for early-staged breast cancer. Int J Surg Oncol.

[20] Fisher S, Gao H, Yasui Y, Dabbs K, Winget M (2015). Survival in stage I-III breast cancer patients by surgical treatment in a publicly funded health care system. Annals of oncology : official journal of the European Society for Medical Oncology / ESMO.

[21] Onitilo AA, Engel JM, Stankowski RV, Doi SA (2014). Survival Comparisons for Breast Conserving Surgery and Mastectomy Revisited: Community Experience and the Role of Radiation Therapy. Clinical medicine & research.

[22] Land LH, Dalton SO, Jensen MB, Ewertz M (2012). Impact of comorbidity on mortality: a cohort study of 62,591 Danish women diagnosed with early breast cancer, 1990-2008. Breast Cancer Res Treat.

[23] Cao JQ, Truong PT, Olivotto IA, Olson R, Coulombe G, Keyes M, Weir L, Gelmon K, Bernstein V, Woods R, Speers C, Tyldesley S (2014). Should women younger than 40 years of age with invasive breast cancer have a mastectomy? 15-year outcomes in a population-based cohort. International journal of radiation oncology, biology, physics.

[24] van der Sangen MJ, van de Wiel FM, Poortmans PM, Tjan-Heijnen VC, Nieuwenhuijzen GA, Roumen RM, Ernst MF, Tutein Nolthenius-Puylaert MC, Voogd AC (2011). Are breast conservation and mastectomy equally effective in the treatment of young women with early breast cancer? Long-term results of a population-based cohort of 1,451 patients aged </= 40 years. Breast Cancer Res Treat.

[25] Ye JC, Yan W, Christos PJ, Nori D, Ravi A (2015). Equivalent Survival With Mastectomy or Breast-conserving Surgery Plus Radiation in Young Women Aged < 40 Years With Early-Stage Breast Cancer: A National Registry-based Stage-by-Stage Comparison. Clinical breast cancer.

[26] Abramovitch R, Marikovsky M, Meir G, Neeman M (1999). Stimulation of tumour growth by wound-derived growth factors. Br J Cancer.

[27] Murthy SM, Goldschmidt RA, Rao LN, Ammirati M, Buchmann T, Scanlon EF (1989). The influence of surgical trauma on experimental metastasis. Cancer.

[28] Bogden AE, Moreau JP, Eden PA (1997). Proliferative response of human and animal tumours to surgical wounding of normal tissues: onset, duration and inhibition. Br J Cancer.

[29] Abramovitch R, Marikovsky M, Meir G, Neeman M (1998). Stimulation of tumour angiogenesis by proximal wounds: spatial and temporal analysis by MRI. Br J Cancer.

[30] Lacy AM, Garcia-Valdecasas JC, Delgado S, Castells A, Taura P, Pique JM, Visa J (2002). Laparoscopy-assisted colectomy versus open colectomy for treatment of non-metastatic colon cancer: a randomised trial. Lancet.

[31] Overgaard M, Nielsen HM, Overgaard J (2007). Is the benefit of postmastectomy irradiation limited to patients with four or more positive nodes, as recommended in international consensus reports? A subgroup analysis of the DBCG 82 b&c randomized trials. Radiother Oncol.

[32] Clarke M, Collins R, Darby S, Davies C, Elphinstone P, Evans V, Godwin J, Gray R, Hicks C, James S, MacKinnon E, McGale P, McHugh T (2005). Effects of radiotherapy and of differences in the extent of surgery for early breast cancer on local recurrence and 15-year survival: an overview of the randomised trials. Lancet.

[33] Ebctcg, McGale P, Taylor C, Correa C, Cutter D, Duane F, Ewertz M, Gray R, Mannu G, Peto R, Whelan T, Wang Y, Wang Z (2014). Effect of radiotherapy after mastectomy and axillary surgery on 10-year recurrence and 20-year breast cancer mortality: meta-analysis of individual patient data for 8135 women in 22 randomised trials. Lancet.

[34] NCCN Clinical Practice Guidelines in Oncology.

[35] He ZY, Wu SG, Zhou J, Li FY, Lin Q, Lin HX, Sun JY (2015). Postmastectomy radiotherapy improves disease-free survival of high risk of locoregional recurrence breast cancer patients with T1-2 and 1 to 3 positive nodes. PloS one.

[36] Huo D, Hou N, Jaskowiak N, Winchester DJ, Winchester DP, Yao K (2015). Use of Postmastectomy Radiotherapy and Survival Rates for Breast Cancer Patients with T1-T2 and One to Three Positive Lymph Nodes. Ann Surg Oncol.

[37] McBride A, Allen P, Woodward W, Kim M, Kuerer HM, Drinka EK, Sahin A, Strom EA, Buzdar A, Valero V, Hortobagyi GN, Hunt KK, Buchholz TA (2014). Locoregional recurrence risk for patients with T1,2 breast cancer with 1-3 positive lymph nodes treated with mastectomy and systemic treatment. International journal of radiation oncology, biology, physics.

[38] Huzarski T, Byrski T, Gronwald J, Gorski B, Domagala P, Cybulski C, Oszurek O, Szwiec M, Gugala K, Stawicka M, Morawiec Z, Mierzwa T, Janiszewska H (2013). Ten-year survival in patients with BRCA1-negative and BRCA1-positive breast cancer. J Clin Oncol.

