# Supplementary material for: Comparative effectiveness study of breast-conserving surgery and mastectomy in the general population: A NCDB analysis
Source: Oncotarget. 2015 Oct 19;6(37):40127–40. doi: 10.18632/oncotarget.5394 (PMC4741884; doi:10.18632/oncotarget.5394)
Supplement: Supplementary file 1 [file oncotarget-06-40127-s001.pdf]

Supplementary Table S1: Univariate analysis of factors associated with overall survival

Supplementary Table S2A: Details of estimates of cox-regression model in the entire population stratified by N-stage

Supplementary Table S2B: Details of estimates of cox-regression model in the population with no/few comorbidities and stratified by N-stage

Supplementary Table S2C: Details of estimates of cox-regression model in the population stratified by N-stage and age. (age>50)

Supplementary Table S2D: Details of estimates of cox-regression model in the population stratified by N-stage and age(age>50)
