# Supplementary material for: Comparative effectiveness study of breast-conserving surgery and mastectomy in the general population: A NCDB analysis
Source: Oncotarget. 2015 Oct 19;6(37):40127–40. doi: 10.18632/oncotarget.5394 (PMC4741884; doi:10.18632/oncotarget.5394)
Supplement: Supplementary file 2 [file oncotarget-06-40127-s002.docx]

| **Suppl Table 1. Univariate analysis of factors associated with overall survival** | | | | |
| --- | --- | --- | --- | --- |
|  | **Univariate analysis** | | | |
| **Variable** | **HR** | **P** | **95%CI(lower)** | **95%CI(upper)** |
| **Facility Type** |  |  |  |  |
| Community Cancer Program | 1.00 |  |  |  |
| Comprehensive Community Cancer Program | 0.85 | <0.001 | 0.80 | 0.90 |
| Academic/Research Program | 0.72 | <0.001 | 0.68 | 0.77 |
| Other specified types of cancer programs | 0.69 | 0.194 | 0.39 | 1.21 |
| **Primary Payor** |  |  |  |  |
| Not Insured | 1.00 |  |  |  |
| Private Insurance | 0.56 | <0.001 | 0.49 | 0.64 |
| Medicaid | 1.11 | 0.166 | 0.96 | 1.30 |
| Medicare | 1.64 | <0.001 | 1.43 | 1.88 |
| Other Government | 0.84 | 0.19 | 0.66 | 1.09 |
| **City type** |  |  |  |  |
| Metropolitan | 1.00 |  |  |  |
| Urban | 1.17 | <0.001 | 1.11 | 1.23 |
| Rural | 1.17 | 0.023 | 1.02 | 1.34 |
| **Dist home to hospital** |  |  |  |  |
| <10_Miles | 1.00 |  |  |  |
| >10_Miles | 0.91 | <0.001 | 0.87 | 0.94 |
| **Median Income 2008-2012*** |  |  |  |  |
| <$47999 | 1.00 |  |  |  |
| $48000+ | 0.69 | <0.001 | 0.66 | 0.71 |
| **Percent No High School Degree 2008-2012**** |  |  |  |  |
| >=13% | 1.00 |  |  |  |
| <13% | 0.76 | <0.001 | 0.74 | 0.79 |
| **Age group** |  |  |  |  |
| <=50 | 1.00 |  |  |  |
| >50 | 1.95 | <0.001 | 1.86 | 2.05 |
| **Race** |  |  |  |  |
| White | 1.00 |  |  |  |
| African American | 1.51 | <0.001 | 1.44 | 1.60 |
| Others | 0.61 | <0.001 | 0.54 | 0.69 |
| **Charlson-Deyo Score** |  |  |  |  |
| 0 | 1.00 |  |  |  |
| 1 | 2.04 | <0.001 | 1.94 | 2.13 |
| 2 | 3.93 | <0.001 | 3.65 | 4.23 |
| **T-stage** |  |  |  |  |
| T1 | 1.00 |  |  |  |
| T2 | 2.43 | <0.001 | 2.34 | 2.52 |
| **N-stage** |  |  |  |  |
| N0 | 1.00 |  |  |  |
| N1 | 1.56 | <0.001 | 1.49 | 1.63 |
| N2 | 2.88 | <0.001 | 2.70 | 3.06 |
| N3 | 4.84 | <0.001 | 4.46 | 5.25 |
| **Stage** |  |  |  |  |
| I | 1.00 |  |  |  |
| II | 2.04 | <0.001 | 1.96 | 2.12 |
| III | 4.11 | <0.001 | 3.89 | 4.34 |
| **Grade** |  |  |  |  |
| I | 1.00 |  |  |  |
| II | 1.46 | <0.001 | 1.37 | 1.55 |
| III | 2.58 | <0.001 | 2.44 | 2.74 |
| **Estrogen receptor** |  |  |  |  |
| Negative | 1.00 |  |  |  |
| Positive | 0.43 | <0.001 | 0.41 | 0.44 |
| **Progesterone receptor** |  |  |  |  |
| Negative | 1.00 |  |  |  |
| Positive | 0.47 | <0.001 | 0.45 | 0.48 |
| **Laterality** |  |  |  |  |
| Right | 1.00 |  |  |  |
| Left | 1.02 | 0.377 | 0.98 | 1.05 |
| **Primary Site** |  |  |  |  |
| Central | 1.00 |  |  |  |
| LIQ | 0.84 | 0.002 | 0.76 | 0.94 |
| LOQ | 0.87 | 0.005 | 0.78 | 0.96 |
| UIQ | 0.74 | <0.001 | 0.67 | 0.81 |
| UOQ | 0.78 | <0.001 | 0.72 | 0.85 |
| Nipple | 1.10 | 0.535 | 0.82 | 1.47 |
| Others | 0.83 | <0.001 | 0.76 | 0.90 |
| **Lymphovascular invasion** |  |  |  |  |
| Negative | 1.00 |  |  |  |
| Positive | 2.19 | <0.001 | 1.97 | 2.43 |
| Unknown | 1.18 | <0.001 | 1.10 | 1.26 |
| **Radiation therapy** |  |  |  |  |
| Negative | 1.00 |  |  |  |
| Positive | 0.43 | <0.001 | 0.41 | 0.45 |
| **Chemotherapy** |  |  |  |  |
| No | 1.00 |  |  |  |
| Yes | 0.88 | <0.001 | 0.85 | 0.91 |
| **Days of Inpatient stay** |  |  |  |  |
| 0 | 1.00 |  |  |  |
| 1 | 1.87 | <0.001 | 1.79 | 1.95 |
| >1 | 2.36 | <0.001 | 2.25 | 2.48 |
| **Surgery (BCS or Mastectomy)** |  |  |  |  |
| BCS+RT | 1.00 |  |  |  |
| Mastectomy alone +/- RT | 2.57 | <0.001 | 2.48 | 2.67 |
| *Indicates the median annual income of the state/area where the patient lived in.  **Indicates the percentage of population that do not have a high school degree in the state/area where the patient lived in.  BCS,breast-conserving surgery; RT, Radiation therapy; LIQ, lower-inner quadrant; LOQ, lower-outer quadrant; UIQ, Upper-inner quadrant; UOQ, Upper-outer quadrant; NS, non-significant; | | | | |
