# Supplementary material for: Comparative effectiveness study of breast-conserving surgery and mastectomy in the general population: A NCDB analysis
Source: Oncotarget. 2015 Oct 19;6(37):40127–40. doi: 10.18632/oncotarget.5394 (PMC4741884; doi:10.18632/oncotarget.5394)
Supplement: Supplementary file 3 [file oncotarget-06-40127-s003.docx]

| **Supplementary table 2a, Details of estimates of cox-regression model in the entire population stratified by N-stage.** | | | | | | | | | | | | |
| --- | --- | --- | --- | --- | --- | --- | --- | --- | --- | --- | --- | --- |
|  | **N0 patients** | | | | **N1 patients** | | | | **N2 patients** | | | |
|  | **HR** | **95%CI lower limit** | **95%CI upper limit** | **P** | **HR** | **95%CI lower limit** | **95%CI upper limit** | **P** | **HR** | **95%CI lower limit** | **95%CI upper limit** | **P** |
| **Facility Type** |  |  |  |  |  |  |  |  |  |  |  |  |
| 1: Community Cancer Program | 1.00 |  |  |  | 1.00 |  |  |  | 1.00 |  |  |  |
| 2: Comprehensive Community Cancer Program | 0.94 | 0.87 | 1.01 | 0.09 | 0.89 | 0.79 | 0.99 | 0.04 | 1.02 | 0.88 | 1.18 | 0.78 |
| 3: Academic/Research Program | 0.81 | 0.75 | 0.88 | <0.001 | 0.84 | 0.74 | 0.95 | 0.01 | 0.83 | 0.71 | 0.98 | 0.02 |
| 9: Other Specified Types Of Cancer Programs | 0.96 | 0.48 | 1.92 | 0.90 | 0.86 | 0.32 | 2.30 | 0.76 | 0.00 | . | . | . |
| **Primary Payor** |  |  |  |  |  |  |  |  |  |  |  |  |
| 0: Not Insured | 1.00 |  |  |  | 1.00 |  |  |  | 1.00 |  |  |  |
| 1: Private Insurance | 0.80 | 0.64 | 1.00 | 0.05 | 0.62 | 0.49 | 0.79 | <0.001 | 0.75 | 0.58 | 0.99 | 0.04 |
| 2: Medicaid | 1.26 | 0.99 | 1.61 | 0.06 | 0.82 | 0.63 | 1.07 | 0.14 | 0.96 | 0.71 | 1.29 | 0.78 |
| 3: Medicare | 1.89 | 1.51 | 2.36 | <0.001 | 1.14 | 0.90 | 1.45 | 0.27 | 1.12 | 0.85 | 1.48 | 0.41 |
| 4: Other Government | 1.27 | 0.90 | 1.81 | 0.18 | 0.79 | 0.50 | 1.26 | 0.32 | 0.56 | 0.28 | 1.14 | 0.11 |
| **City Type** |  |  |  |  |  |  |  |  |  |  |  |  |
| Metropolitan | 1.00 |  |  |  | 1.00 |  |  |  | 1.00 |  |  |  |
| Urban | 0.98 | 0.90 | 1.06 | 0.60 | 1.02 | 0.91 | 1.15 | 0.72 | 0.96 | 0.82 | 1.11 | 0.56 |
| Rural | 0.92 | 0.76 | 1.10 | 0.35 | 0.80 | 0.60 | 1.05 | 0.11 | 0.88 | 0.63 | 1.25 | 0.48 |
| **Distance** |  |  |  |  |  |  |  |  |  |  |  |  |
| <10_Miles | 1.00 |  |  |  | 1.00 |  |  |  | 1.00 |  |  |  |
| >10_Miles | 0.92 | 0.88 | 0.98 | <0.001 | 1.05 | 0.97 | 1.13 | 0.24 | 1.11 | 1.00 | 1.22 | 0.06 |
| **Median Income Quartiles 2008-2012** | | |  |  |  |  |  |  |  |  |  |  |
| <$47999 | 1.00 |  |  |  | 1.00 |  |  |  | 1.00 |  |  |  |
| $48000+ | 0.82 | 0.78 | 0.88 | <0.001 | 0.88 | 0.81 | 0.97 | 0.01 | 0.87 | 0.78 | 0.98 | 0.02 |
| **Percent No High School Degree 2008-2012** | | |  |  |  |  |  |  |  |  |  |  |
| >=13% | 1.00 |  |  |  | 1.00 |  |  |  | 1.00 |  |  |  |
| <13% | 1.04 | 0.98 | 1.10 | 0.22 | 1.05 | 0.96 | 1.14 | 0.29 | 1.00 | 0.90 | 1.12 | 0.97 |
| **Age Group** |  |  |  |  |  |  |  |  |  |  |  |  |
| <=60 | 1.00 |  |  |  | 1.00 |  |  |  | 1.00 |  |  |  |
| >60 | 1.38 | 1.28 | 1.50 | <0.001 | 1.17 | 1.06 | 1.30 | <0.001 | 1.18 | 1.04 | 1.33 | 0.01 |
| **Race** |  |  |  |  |  |  |  |  |  |  |  |  |
| White | 1.00 |  |  |  | 1.00 |  |  |  | 1.00 |  |  |  |
| African American | 1.08 | 1.00 | 1.17 | 0.05 | 1.19 | 1.07 | 1.33 | <0.001 | 1.26 | 1.11 | 1.43 | <0.001 |
| Others | 0.59 | 0.50 | 0.71 | <0.001 | 0.69 | 0.54 | 0.87 | <0.001 | 0.77 | 0.59 | 1.00 | 0.05 |
| **Charlson-Deyo Score** | |  |  |  |  |  |  |  |  |  |  |  |
| 0 | 1.00 |  |  |  | 1.00 |  |  |  | 1.00 |  |  |  |
| 1 | 1.54 | 1.45 | 1.64 | <0.001 | 1.45 | 1.32 | 1.59 | <0.001 | 1.33 | 1.18 | 1.50 | <0.001 |
| 2 | 2.48 | 2.24 | 2.74 | <0.001 | 2.21 | 1.91 | 2.56 | <0.001 | 1.78 | 1.46 | 2.16 | <0.001 |
| **T-Stage** |  |  |  |  |  |  |  |  |  |  |  |  |
| T1 | 1.00 |  |  |  | 1.00 |  |  |  | 1.00 |  |  |  |
| T2 | 1.89 | 1.79 | 1.99 | <0.001 | 1.67 | 1.55 | 1.80 | <0.001 | 1.26 | 1.13 | 1.39 | <0.001 |
| **Grade** |  |  |  |  |  |  |  |  |  |  |  |  |
| I | 1.00 |  |  |  |  |  |  |  |  |  |  |  |
| II | 1.17 | 1.08 | 1.25 | <0.001 | 1.44 | 1.25 | 1.66 | <0.001 | 1.44 | 1.11 | 1.86 | 0.01 |
| III | 1.46 | 1.35 | 1.59 | <0.001 | 2.01 | 1.74 | 2.33 | <0.001 | 1.97 | 1.52 | 2.54 | <0.001 |
| **Estrogen Receptor** |  |  |  |  |  |  |  |  |  |  |  |  |
| Negative | 1.00 |  |  |  | 1.00 |  |  |  | 1.00 |  |  |  |
| Positive | 0.60 | 0.55 | 0.65 | <0.001 | 0.46 | 0.41 | 0.53 | <0.001 | 0.62 | 0.54 | 0.72 | <0.001 |
| **Progesterone Receptor** | |  |  |  |  |  |  |  |  |  |  |  |
| Negative | 1.00 |  |  |  | 1.00 |  |  |  | 1.00 |  |  |  |
| Positive | 0.79 | 0.74 | 0.86 | <0.001 | 0.78 | 0.69 | 0.88 | <0.001 | 0.64 | 0.55 | 0.74 | <0.001 |
| **Lymphovascular Invasion** | |  |  |  |  |  |  |  |  |  |  |  |
| Negative | 1.00 |  |  |  | 1.00 |  |  |  | 1.00 |  |  |  |
| Positive | 1.35 | 1.13 | 1.63 | <0.001 | 1.46 | 1.20 | 1.77 | <0.001 | 1.18 | 0.91 | 1.52 | 0.21 |
| Unknown | 0.95 | 0.87 | 1.03 | 0.20 | 1.12 | 0.96 | 1.31 | 0.14 | 1.00 | 0.80 | 1.25 | 1.00 |
| **Sites** |  |  |  |  |  |  |  |  |  |  |  |  |
| Central | 1.00 |  |  |  | 1.00 |  |  |  | 1.00 |  |  |  |
| LIQ | 1.06 | 0.92 | 1.22 | 0.41 | 0.99 | 0.80 | 1.21 | 0.90 | 1.03 | 0.78 | 1.36 | 0.83 |
| LOQ | 1.04 | 0.90 | 1.19 | 0.62 | 1.00 | 0.84 | 1.20 | 0.98 | 0.93 | 0.73 | 1.18 | 0.55 |
| UIQ | 0.99 | 0.87 | 1.13 | 0.91 | 0.97 | 0.81 | 1.16 | 0.72 | 1.11 | 0.86 | 1.43 | 0.43 |
| UOQ | 0.96 | 0.85 | 1.08 | 0.50 | 0.87 | 0.74 | 1.01 | 0.06 | 1.04 | 0.85 | 1.27 | 0.71 |
| Nipple | 1.06 | 0.69 | 1.63 | 0.80 | 1.08 | 0.65 | 1.81 | 0.76 | 2.02 | 1.08 | 3.76 | 0.03 |
| Others | 1.01 | 0.89 | 1.13 | 0.92 | 0.94 | 0.81 | 1.10 | 0.43 | 0.97 | 0.79 | 1.19 | 0.74 |
| **Chemotherapy** |  |  |  |  |  |  |  |  |  |  |  |  |
| No | 1.00 |  |  |  | 1.00 |  |  |  | 1.00 |  |  |  |
| Yes | 0.59 | 0.55 | 0.62 | <0.001 | 0.40 | 0.37 | 0.44 | <0.001 | 0.40 | 0.35 | 0.46 | <0.001 |
| **Days Of Inpatient Stay** | |  |  |  |  |  |  |  |  |  |  |  |
| 0 | 1.00 |  |  |  | 1.00 |  |  |  | 1.00 |  |  |  |
| 1 | 1.10 | 1.03 | 1.18 | 0.01 | 0.95 | 0.87 | 1.04 | 0.29 | 1.03 | 0.91 | 1.15 | 0.66 |
| >1 | 1.20 | 1.11 | 1.30 | <0.001 | 1.15 | 1.04 | 1.28 | 0.01 | 1.10 | 0.96 | 1.25 | 0.18 |
| **Surgery type** |  |  |  |  |  |  |  |  |  |  |  |  |
| BCS+RT | 1.00 |  |  |  | 1.00 |  |  |  | 1.00 |  |  |  |
| Mastectomy_alone | 1.40 | 1.31 | 1.50 | <0.001 | 1.44 | 1.31 | 1.58 | <0.001 | 1.64 | 1.42 | 1.88 | <0.001 |
| Mastectomy+RT | 1.52 | 1.26 | 1.83 | <0.001 | 1.33 | 1.17 | 1.51 | <0.001 | 1.12 | 1.00 | 1.27 | 0.05 |
