# Supplementary material for: Comparative effectiveness study of breast-conserving surgery and mastectomy in the general population: A NCDB analysis
Source: Oncotarget. 2015 Oct 19;6(37):40127–40. doi: 10.18632/oncotarget.5394 (PMC4741884; doi:10.18632/oncotarget.5394)
Supplement: Supplementary file 5 [file oncotarget-06-40127-s005.docx]

| **Supplementary table 2c, Details of estimates of cox-regression model in the population stratified by N-stage and age.(age<=50)** | | | | | | | | | | | | |
| --- | --- | --- | --- | --- | --- | --- | --- | --- | --- | --- | --- | --- |
|  | **N0 patients** | | | | **N1 patients** | | | | **N2 patients** | | | |
|  | **HR** | **95%CI lower limit** | **95%CI upper limit** | **P** | **HR** | **95%CI lower limit** | **95%CI upper limit** | **P** | **HR** | **95%CI lower limit** | **95%CI upper limit** | **P** |
| **Facility Type** |  |  |  |  |  |  |  |  |  |  |  |  |
| 1: Community Cancer Program | 1.00 |  |  |  | 1.00 |  |  |  | 1.00 |  |  |  |
| 2: Comprehensive Community Cancer Program | 1.09 | 0.86 | 1.38 | 0.48 | 0.91 | 0.69 | 1.20 | 0.51 | 0.78 | 0.58 | 1.05 | 0.10 |
| 3: Academic/Research Program | 0.95 | 0.74 | 1.23 | 0.70 | 0.91 | 0.68 | 1.21 | 0.51 | 0.69 | 0.50 | 0.96 | 0.03 |
| 9: Other Specified Types Of Cancer Programs | 0.00 | . | . | . | 1.59 | 0.22 | 11.71 | 0.65 | 0.00 | 0.00 | . | 1.00 |
| **Primary Payor** |  |  |  |  |  |  |  |  |  |  |  |  |
| 0: Not Insured | 1.00 |  |  |  | 1.00 |  |  |  | 1.00 |  |  |  |
| 1: Private Insurance | 0.93 | 0.62 | 1.40 | 0.73 | 0.55 | 0.39 | 0.78 | <0.001 | 0.62 | 0.41 | 0.94 | 0.02 |
| 2: Medicaid | 1.32 | 0.86 | 2.04 | 0.21 | 0.77 | 0.52 | 1.13 | 0.18 | 0.89 | 0.57 | 1.41 | 0.62 |
| 3: Medicare | 2.43 | 1.52 | 3.90 | <0.001 | 0.75 | 0.44 | 1.29 | 0.30 | 1.11 | 0.63 | 1.97 | 0.72 |
| 4: Other Government | 1.58 | 0.79 | 3.16 | 0.20 | 0.88 | 0.42 | 1.84 | 0.73 | 0.95 | 0.33 | 2.78 | 0.93 |
| **City Type** |  |  |  |  |  |  |  |  |  |  |  |  |
| Metropolitan | 1.00 |  |  |  | 1.00 |  |  |  | 1.00 |  |  |  |
| Urban | 1.03 | 0.82 | 1.29 | 0.83 | 1.27 | 0.97 | 1.66 | 0.08 | 1.14 | 0.83 | 1.57 | 0.41 |
| Rural | 0.83 | 0.45 | 1.53 | 0.55 | 0.99 | 0.50 | 1.96 | 0.97 | 0.83 | 0.38 | 1.82 | 0.64 |
| **Distance** |  |  |  |  |  |  |  |  |  |  |  |  |
| <10_Miles | 1.00 |  |  |  | 1.00 |  |  |  | 1.00 |  |  |  |
| >10_Miles | 1.11 | 0.95 | 1.29 | 0.18 | 1.06 | 0.89 | 1.27 | 0.51 | 1.06 | 0.85 | 1.32 | 0.60 |
| **Median Income Quartiles 2008-2012** | |  |  |  |  |  |  |  |  |  |  |  |
| <$47999 | 1.00 |  |  |  | 1.00 |  |  |  | 1.00 |  |  |  |
| $48000+ | 0.70 | 0.59 | 0.84 | <0.001 | 0.91 | 0.73 | 1.12 | 0.35 | 1.05 | 0.81 | 1.35 | 0.71 |
| **Percent No High School Degree 2008-2012** | | |  |  |  |  |  |  |  |  |  |  |
| >=13% | 1.00 |  |  |  | 1.00 |  |  |  | 1.00 |  |  |  |
| <13% | 1.19 | 1.00 | 1.41 | 0.05 | 1.09 | 0.89 | 1.33 | 0.42 | 0.89 | 0.69 | 1.13 | 0.34 |
| **Race** |  |  |  |  |  |  |  |  |  |  |  |  |
| White | 1.00 |  |  |  | 1.00 |  |  |  | 1.00 |  |  |  |
| African American | 1.24 | 1.03 | 1.49 | 0.02 | 1.12 | 0.90 | 1.38 | 0.31 | 1.16 | 0.90 | 1.49 | 0.27 |
| Others | 0.78 | 0.54 | 1.13 | 0.19 | 0.61 | 0.38 | 0.99 | 0.04 | 0.91 | 0.56 | 1.47 | 0.70 |
| **Charlson-Deyo Score** |  |  |  |  |  |  |  |  |  |  |  |  |
| 0 | 1.00 |  |  |  | 1.00 |  |  |  | 1.00 |  |  |  |
| 1 | 1.32 | 1.03 | 1.69 | 0.03 | 1.39 | 1.04 | 1.85 | 0.03 | 1.25 | 0.91 | 1.73 | 0.17 |
| 2 | 2.28 | 1.44 | 3.61 | <0.001 | 1.68 | 0.91 | 3.12 | 0.10 | 3.30 | 1.74 | 6.26 | <0.001 |
| **T-Stage** |  |  |  |  |  |  |  |  |  |  |  |  |
| T1 | 1.00 |  |  |  | 1.00 |  |  |  | 1.00 |  |  |  |
| T2 | 1.80 | 1.55 | 2.09 | <0.001 | 1.95 | 1.63 | 2.33 | <0.001 | 1.20 | 0.96 | 1.49 | 0.11 |
| **Grade** |  |  |  |  |  |  |  |  |  |  |  |  |
| I | 1.00 |  |  |  | 1.00 |  |  |  | 1.00 |  |  |  |
| II | 1.75 | 1.27 | 2.42 | <0.001 | 1.54 | 0.95 | 2.51 | 0.08 | 1.21 | 0.63 | 2.34 | 0.57 |
| III | 2.10 | 1.50 | 2.95 | <0.001 | 2.55 | 1.57 | 4.14 | <0.001 | 1.62 | 0.84 | 3.10 | 0.15 |
| **Estrogen Receptor** |  |  |  |  |  |  |  |  |  |  |  |  |
| Negative | 1.00 |  |  |  | 1.00 |  |  |  | 1.00 |  |  |  |
| Positive | 0.50 | 0.38 | 0.65 | <0.001 | 0.43 | 0.32 | 0.58 | <0.001 | 0.44 | 0.31 | 0.62 | <0.001 |
| **Progesterone Receptor** |  |  |  |  |  |  |  |  |  |  |  |  |
| Negative | 1.00 |  |  |  | 1.00 |  |  |  | 1.00 |  |  |  |
| Positive | 0.68 | 0.52 | 0.90 | 0.01 | 0.62 | 0.46 | 0.85 | <0.001 | 0.71 | 0.50 | 1.01 | 0.05 |
| **Lymphovascular Invasion** | |  |  |  |  |  |  |  |  |  |  |  |
| Negative | 1.00 |  |  |  | 1.00 |  |  |  | 1.00 |  |  |  |
| Positive | 1.52 | 0.95 | 2.43 | 0.08 | 2.71 | 1.48 | 4.96 | <0.001 | 1.72 | 0.86 | 3.43 | 0.13 |
| Unknown | 0.76 | 0.59 | 0.97 | 0.03 | 2.16 | 1.28 | 3.65 | <0.001 | 1.73 | 0.93 | 3.20 | 0.08 |
| **Sites** |  |  |  |  |  |  |  |  |  |  |  |  |
| Central | 1.00 |  |  |  | 1.00 |  |  |  | 1.00 |  |  |  |
| LIQ | 0.94 | 0.60 | 1.49 | 0.81 | 1.76 | 0.95 | 3.29 | 0.07 | 0.57 | 0.30 | 1.10 | 0.10 |
| LOQ | 1.21 | 0.78 | 1.87 | 0.39 | 1.77 | 0.98 | 3.20 | 0.06 | 0.61 | 0.35 | 1.07 | 0.09 |
| UIQ | 0.87 | 0.58 | 1.32 | 0.52 | 1.74 | 0.97 | 3.11 | 0.06 | 0.59 | 0.33 | 1.04 | 0.07 |
| UOQ | 0.76 | 0.52 | 1.13 | 0.18 | 1.27 | 0.73 | 2.18 | 0.40 | 0.67 | 0.42 | 1.07 | 0.09 |
| Nipple | 1.54 | 0.47 | 5.09 | 0.48 | 2.21 | 0.50 | 9.76 | 0.30 | 0.00 | 0.00 | . | 1.00 |
| Others | 0.97 | 0.65 | 1.44 | 0.88 | 1.60 | 0.93 | 2.76 | 0.09 | 0.66 | 0.41 | 1.06 | 0.09 |
| **Chemotherapy** |  |  |  |  |  |  |  |  |  |  |  |  |
| No | 1.00 |  |  |  | 1.00 |  |  |  | 1.00 |  |  |  |
| Yes | 0.96 | 0.79 | 1.17 | 0.70 | 0.51 | 0.36 | 0.74 | <0.001 | 0.62 | 0.35 | 1.11 | 0.11 |
| **Days Of Inpatient Stay** |  |  |  |  |  |  |  |  |  |  |  |  |
| 0 | 1.00 |  |  |  | 1.00 |  |  |  | 1.00 |  |  |  |
| 1 | 1.08 | 0.88 | 1.32 | 0.45 | 1.00 | 0.82 | 1.22 | 0.98 | 1.62 | 1.28 | 2.05 | <0.001 |
| >1 | 1.05 | 0.82 | 1.34 | 0.69 | 0.96 | 0.74 | 1.24 | 0.75 | 1.39 | 1.03 | 1.87 | 0.03 |
| **Surgery type** |  |  |  |  |  |  |  |  |  |  |  |  |
| BCS+RT | 1.00 |  |  |  | 1.00 |  |  |  | 1.00 |  |  |  |
| Mastectomy_alone | 1.42 | 1.16 | 1.74 | <0.001 | 1.13 | 0.90 | 1.43 | 0.29 | 1.20 | 0.87 | 1.67 | 0.27 |
| Mastectomy+RT | 1.70 | 1.15 | 2.50 | 0.01 | 1.23 | 0.96 | 1.57 | 0.10 | 0.92 | 0.72 | 1.17 | 0.51 |
